# Enhancing production efficiency through optimizing plant density in maize–soybean strip intercropping

**DOI:** 10.3389/fpls.2024.1473786

**Published:** 2024-10-11

**Authors:** Guanghao Li, Yuwen Liang, Qiannan Liu, Jinghan Zeng, Qingming Ren, Jian Guo, Fei Xiong, Dalei Lu

**Affiliations:** ^1^ Jiangsu Key Laboratory of Crop Genetics and Physiology/Jiangsu Co-Innovation Center for Modern Production Technology of Grain Crops, Agricultural College of Yangzhou University, Yangzhou, China; ^2^ Joint International Research Laboratory of Agriculture and Agri-Product Safety, the Ministry of Education of China, Yangzhou University, Yangzhou, China; ^3^ College of Bioscience and Biotechnology, Yangzhou University, Yangzhou, China

**Keywords:** maize-soybean strip intercropping, density, yield, nitrogen use efficiency, land equivalent ratio, economic benefit

## Abstract

**Introduction:**

Due to limited arable land resources, intercropping has emerged as an efficient and sustainable production method for increasing total grain yield per unit land area. Maize–soybean strip intercropping (MSSI) technology is being widely promoted and applied across China. However, the combination of optimal density for achieving higher production efficiency of both soybean and maize remains unclear. The objective of this study was to evaluate the differences in yield, economic benefits, land, and nitrogen (N) efficiency in MSSI systems under different densities.

**Methods:**

Five maize/soybean density combinations (67,500/97,500 plants ha^−1^, D1; 67,500/120,000 plants ha^−1^, D2; 67,500/142,500 plants ha^−1^, D3; 60,000/142,500 plants ha^−1^, D4; 52,500/142,500 plants ha^−1^, D5) were set under the same N input in the field experiment.

**Results and discussion:**

The results demonstrated that optimizing the density in the intercropping system could enhance production efficiency. Increasing the density of soybean and maize significantly increased the total grain yield (D3 > D2 > D1 > D4 > D5). The D3 treatment, exhibiting the best comprehensive performance, also promoted increases in leaf area index, dry matter accumulation, and N absorption and utilization. Path analysis indicated that density had the most substantial impact on maize yield, while grain number had the greatest influence on soybean yield, with contribution rates of 49.7% and 61.0%, respectively. These results provide valuable insights into optimal field density for summer planting in MSSI, facilitating its wider adoption.

## 1 Introduction

In recent decades, as the global population has increased and temperatures have risen, the focus of agriculture has remained on boosting total food production (Moritz and Agudo, 2013; Tyczewska et al., 2018). China, with a population of 1.4 billion, must enhance its food production capacity to feed nearly 20% of the world’s population with only 7% of arable land (Li et al., 2022a). The limitation of China’s cultivated land area has prevented it from achieving higher output simply by expanding the planting area of specific crops, as this would inevitably sacrifice the planting area of other crops (Li et al., 2014; Liang et al., 2023). Therefore, increasing the yield per unit of land on the limited cultivated land area has become an urgent challenge in agricultural production, both in China and globally.

Intercropping has been demonstrated as a sustainable production method that can alleviate the competition for limited land resources among crops. By rationally selecting two or more crops, the intercropping system achieves higher yield than single cropping, which is of great significance to agricultural production (Li et al., 2023a). The land equivalent ratio (LER) is a critical indicator for assessing the land-use efficiency of intercropping systems. A recent meta-analysis revealed that intercropping crops can achieve an average LER of 1.23, indicating that monoculture requires an additional 23% of farmland to obtain the same yield as intercropping. This productive model is popular in countries with restricted arable land resources (Li et al., 2023a). Maize–soybean strip intercropping (MSSI) has been effectively integrated into China’s agricultural production, providing a viable strategy for ensuring national food security (Liu and Yang, 2024). Previous studies have shown the mechanisms by which MSSI increases system yield. Due to the adjustment of row spacing and plant density, the density of maize (or soybean) under MSSI was similar to that of conventional cultivation. In addition, compared with the monoculture cultivation mode, maize in the MSSI planting system had more sufficient light, root growth space, and soil resources, resulting in a more significant border row effects, which were conducive to higher yield (Du et al., 2018; Zhang et al., 2020a; Shen et al., 2023).

Planting density is crucial for boosting crop yield. Nonetheless, overly high planting density could impede the regular growth of crops (Tokatlidis et al., 2010). Previous studies demonstrated that increasing density enhances competition for light resources, consequently impacting the accumulation of dry matter in leaves and stems. This results in a decrease in dry matter allocated to harvest organs and ultimately reduces yield (Liu et al., 2015; Zhang et al., 2020a). In contrast, intercropping crops could increase the utilization efficiencies of water, light, soil nutrients, and other growth resources, indicating that intercropping crops have a stronger ability to tolerate high density (Hauggaard-Nielsen et al., 2006; Zhang et al., 2020b; Kong et al., 2024). Previous studies suggested that the higher the density of intercropping crops, the greater the intercropping advantage in maize–soybean intercropping (Zhu et al., 2015). This is because planting density in the intercropping system affects the competitive advantage between crops, with higher densities often leading to a dominant competitive position for the crops involved (Ren et al., 2016). However, some studies have shown that excessively high maize density can create shading effects on soybeans, reduce biomass accumulation, and severely limit soybean yield (Liu et al., 2017; Xia et al., 2019). In contrast, maize can achieve higher system yield at medium densities (Yang et al., 2022; Wang et al., 2023b). A study on intercropping maize with multiple varieties of peas showed that legumes could achieve higher output when the density of maize was the same and the density of legumes reached 75% of that of monoculture (Nurgi et al., 2023). Currently, there is still uncertainty regarding the final yield formation when both maize and soybean densities are adjusted simultaneously.

Optimal plant density varies across different regions due to climate variables such as precipitation, solar radiation, temperature, and soil properties (Kang et al., 2009; Ren et al., 2016; Ni and Vellend, 2024). Most studies on MSSI have concentrated on elucidating the natural advantages of the planting system (Fu et al., 2023; Shen et al., 2023; Wang et al., 2023b). However, few studies have explored the optimal density combination for achieving higher yields in MSSI. Therefore, this study established specific combinations of three maize densities and three soybean densities to address this gap. The objectives were: (1) to clarify the optimal density combination in MSSI; (2) to quantify the grain yield, yield components, economic benefits, dry matter accumulation, N accumulation, and utilization efficiency of soybean and maize under different combinations; and (3) to evaluate the differences of each crop’s yield components on the overall yield in MSSI using path analysis. The results will provide a theoretical reference for optimizing field density in MSSI, thereby improving grain productivity and achieving sustainable development.

## 2 Materials and methods

### 2.1 Test site and materials

**Figure 1:**
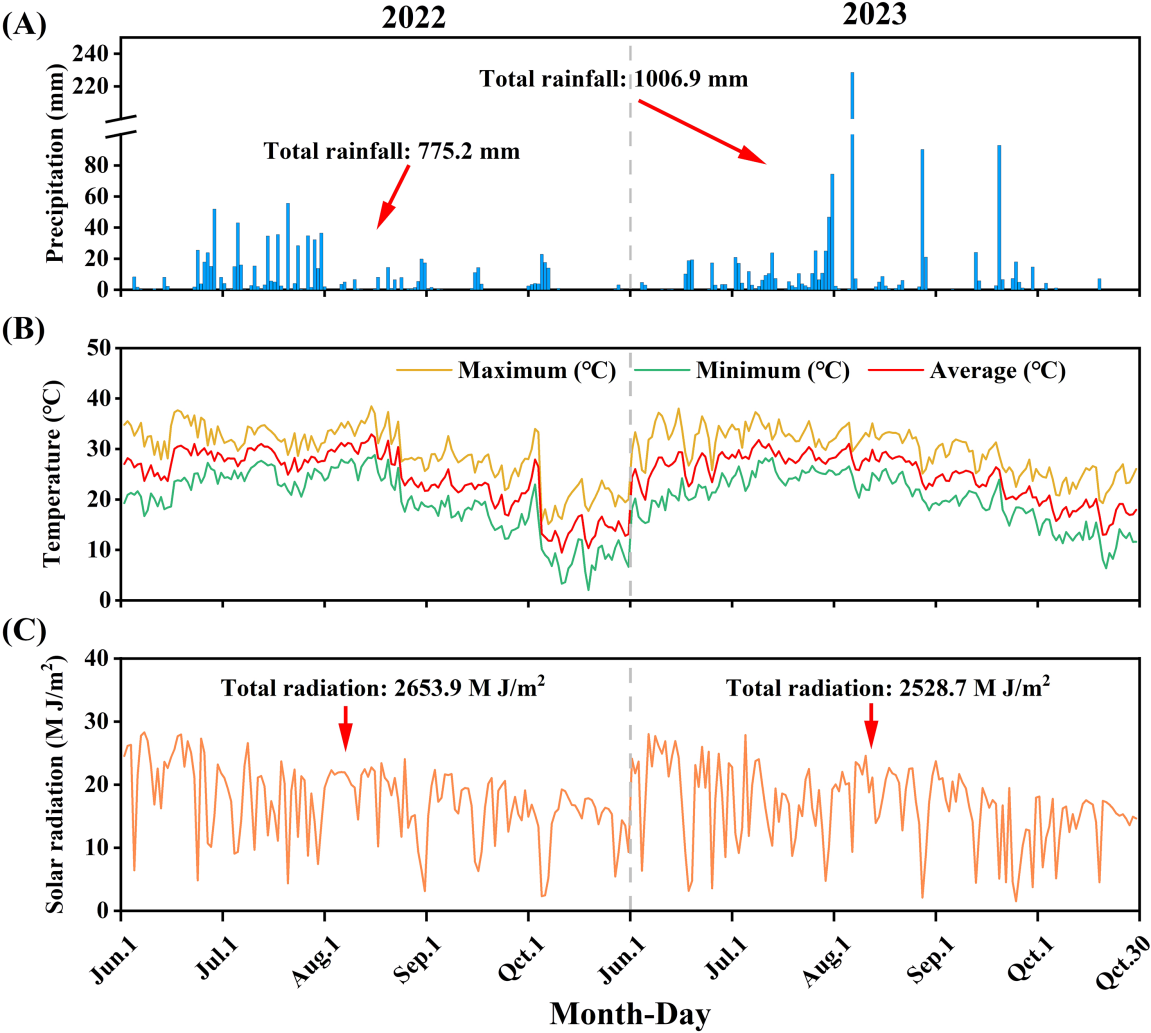
The distribution of precipitation **(A)**, temperature **(B)**, and solar radiation **(C)** in the MSSI system during the experimental periods of 2022 and 2023. The data for rainfall and solar radiation shown covers the period from 1 June 1 to 30 October.

A 2-year experiment (from 2022 to 2023) was conducted at the experimental station of the Suqian (wheat and maize or soybean rotation) promotion demonstration base (34°00′, 118°17′E) in Jiangsu Province, China, which experiences a temperate continental monsoon climate. The soil at the test site was sandy loam, and the area had been used for wheat and maize rotation for several years. During the experimental period from June to October in 2022 and 2023, the rainfall recorded was 775.2 mm and 1,006.9 mm, with average temperatures of 24.1°C and 24.7°C, respectively (**Figure 1**). The soil pH, organic matter, total N, alkaline hydrolysis nitrogen, available phosphorus, and available potassium in the topsoil (0–20 cm) prior to the experiment were 8.3, 13.92 g kg^−1^, 1.01 g kg^−1^, 82.21 mg kg^−1^, 26.16 mg kg^−1^, and 123.50 mg kg^−1^, respectively.

### 2.2 Experimental design

**Figure 2:**
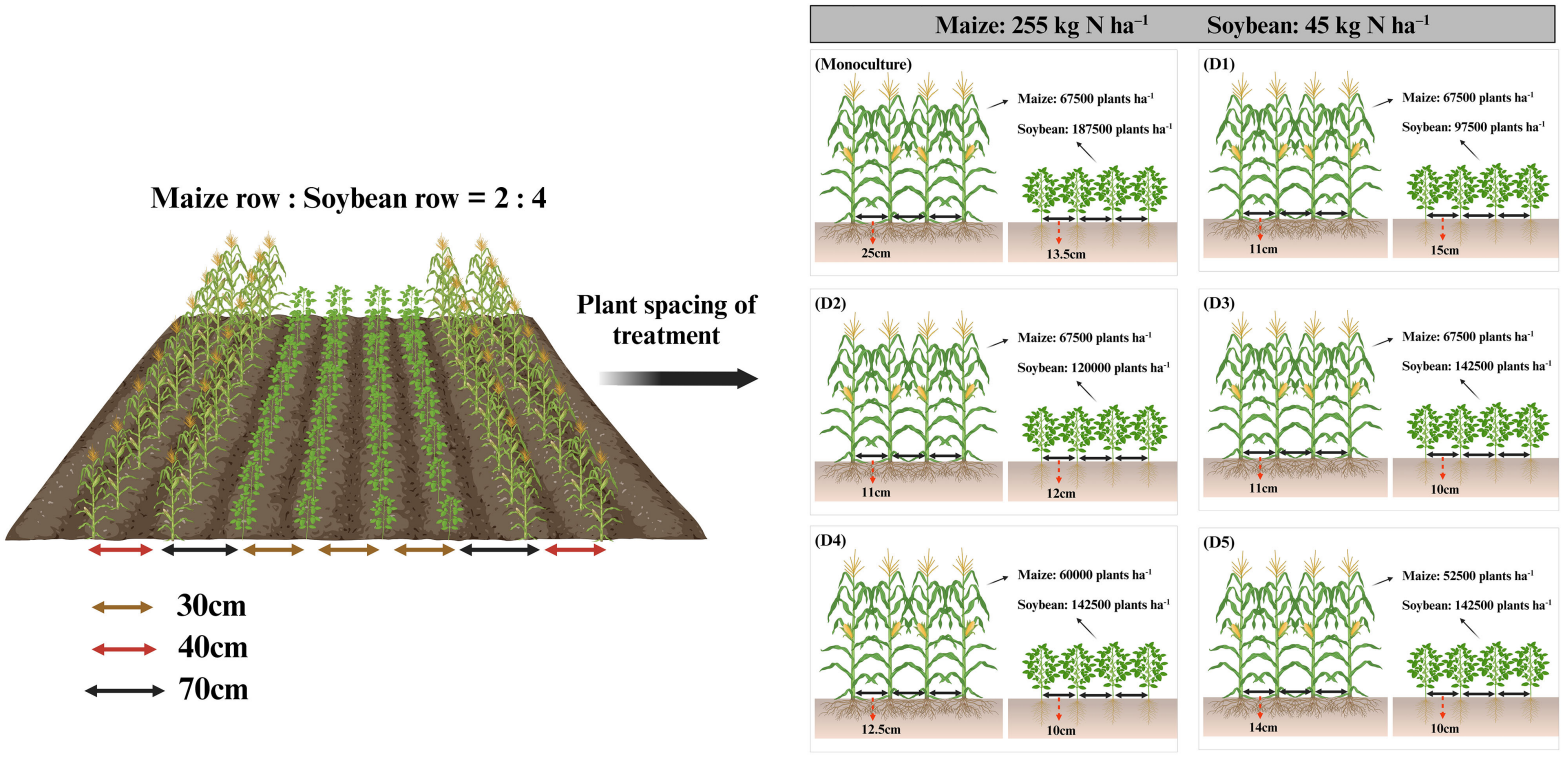
The diagram showing plant spacing and nitrogen input for different treatments in the field.

One single-factor completely randomized design experiments were conducted using maize (Jiangyu 877) and soybean (Xudou 18) as test crops, Jiangyu 877 is a medium-maturing, semicompact maize variety with a growth period of 102 days and a suitable planting density of 5.25 × 10^4^ plants ha^−1^ to 6.75 × 10^4^ plants ha^−1^. Xudou 18 is a soybean variety with a growth period of 104 days and a planting density ranging from 15 × 10^4^ plants ha^−1^ to 22.5 × 10^4^ plants ha^−1^. Both varieties are widely planted in Jiangsu Province. In the experiment, five treatments were established (D1, D2, D3, D4, and D5), representing maize (soybean) planting densities of 6.75 × 10^4^ (9.75 × 10^4^) plants ha^−1^, 6.75 × 10^4^ (12 × 10^4^) plants ha^−1^, 6.75 × 10^4^ (14.25 × 10^4^) plants ha^−1^, 6.0 × 10^4^ (14.25 × 10^4^) plants ha^−1^, and 5.25 × 10^4^ (14.25 × 10^4^) plants ha^−1^, respectively, with the conventional monoculture density serving as the control (**Figure 2**). Each treatment plot had an area of 20 m × 8.8 m and consisted of three planting belts (Each planting belt adopted an intercropping mode of four rows of soybean and two rows of maize, with a row spacing of 40 cm for maize and 30 cm for soybean. The spacing between the soybean belt and maize belt was 70 cm). Soybean and maize were sown at the same time on 23 June and harvested on 8 October each year.

Fertilization and sowing were carried out simultaneously, without any subsequent topdressing. Maize received a one-time application of slow-released fertilizer (N/P_2_O_5_/K_2_O = 27%/9%/9%) applied between two rows of maize. Soybean was fertilized with a compound fertilizer (N/P_2_O_5_/K_2_O = 15%/15%/15%) applied between the first and third rows of the soybean belt. The N application rates of maize and soybean were 255 kg ha^−1^ and 45 kg ha^−1^, respectively. All fertilizers were provided by Jiangsu Zhongdong Fertilizer Co. Ltd. (Changzhou, Jiangsu, China). The rest of the field management practices followed conventional methods.

### 2.3 Sampling and measurement

#### 2.3.1 Dry matter and N accumulation

Plant samples were collected at various growth stages: maize at the tasseling (VT), milk (R3), and maturity (R6) stages; and soybean at the first flowering date (R1), beginning pod (R4), and maturity (R8). Three plants were sampled from each plot. For maize, plant samples were separated into stem and leaf at VT, and into five organs (stem, leaf, husk, cob, and grain) at R3 and R6. Soybean samples were separated into stem and leaf at R1, three organs (stem, leaf, and pods) at R4, and three organs (stem, pods, and grain) at R8. After sampling, all samples were placed in a 105°C oven for 30 min, then dried to a constant weight at 80°C, and weighed. After weighing, all samples were crushed by a cyclone sample mill with a fine mesh (0.5 mm). Subsequently, N concentrations in different samples were determined using the standard Kjeldahl method. The total N accumulation for the whole maize (or soybean) plant was calculated as follows:


Total N accumulation=(Dry matter weight of each plant sample×N concentration

#### 2.3.2 Yield calculation

At the maturity stage, three experimental sites, each measuring 5 m × 2.7 m, were selected in each plot to measure the effective plant number (EP) of maize and soybean. Subsequently, 10 maize ears and 10 soybeans (one plant per interval of five plants) were selected as samples from each plot. After natural drying, the number of grains per plant for both maize and soybean was recorded (GN). Grain moisture content was measured using a grain moisture meter after threshing, with the average of three repeated measurements taken as the actual moisture content. Finally, 1,000 grains of maize and 100 grains of soybean were randomly selected for weighing, repeated three times, and converted into 1,000-grain weight (TKW) for maize and 100-grain weight (HKW) for soybean, both adjusted to standard moisture contents (14% for maize and 13% for soybean). The final yield calculation was as follows:


$$Yield_{M}=EP\times GN\times TKW\times 10^{-6}$$

$$Yield_{S}=EP\times GN\times HKW\times 10^{-5}$$

$$Total\;Yield=Yield_{M}+Yield_{S}$$

#### 2.3.3 Leaf area index

Three representative and uniformly growing plants were tagged at the VT and R3 stages for maize, and at the R1 and R4 stages for soybean. The length and maximum width of each maize leaf were measured. The calculation formula per plant is as follows (Fu et al., 2023):


Leaf area of maize=Length×Width×0.75

The leaf area of soybean per plant was measured using Li-Cor 3050 leaf area meter, produced by LI-COR Company, USA.


LAI of maize(soybean)=(Leaf area per plant×Plant number per plot)/plot area

#### 2.3.4 Land equivalent ratio

LER and partial land equivalent ratio (PLER) were used to evaluate the productivity of intercropped arable lands. The LER and PLER equations were based on the study of Zhang et al. (2021).


$$\text{LER}=PLER_{M}+PLER_{S}=\frac{Y_{IM}}{Y_{M}}+\frac{Y_{IS}}{Y_{S}}$$

LER < 1.0 indicates that intercropping is inferior to monoculture, while LER > 1.0 indicates that intercropping is superior to monoculture. Where *Y*_M_ and *Y*_S_ represent the yields of maize and soybeans in monoculture, respectively, and *Y*_IM_ and *Y*_IS_ represent the yields of maize and soybeans in intercropping.

#### 2.3.5 Actual yield loss

The actual yield loss (AYL) was calculated with reference to Liu et al. (2023).


$$\text{AYL}=AYL_{M}+AYL_{S}=\left(\frac{Y_{IM}}{Y_{M}}\times \frac{P_{M}}{P_{IM}}-1\right)+\left(\frac{Y_{IS}}{Y_{S}}\times \frac{P_{S}}{P_{IS}}-1\right)$$

*P*_M_ and *P*_S_ represent the planting proportion of maize and soybean in monoculture, respectively (both of which are 1), and *P*_IM_ and *P*_IS_ represent the planting proportions of maize and soybean in intercropping, respectively (both of which are 0.5). AYL_X_ > 0 indicates that the relative yield of the intercropped crop *x* is higher than that of monoculture, while AYL_X_ < 0 indicates that the relative yield of the intercropped *x* crop is lower than that of monoculture.

#### 2.3.6 Nitrogen use efficiency

The nitrogen use efficiency (NUE) was calculated with reference to Chen et al. (2017).


$$\text{NUE}\left(\%\right)=\frac{N_{IM}+N_{IS}-N_{IM0}-N_{IS0}}{N_{M}+N_{S}}$$

Where *N*_IM_ (or *N*_IS_) is the total N accumulation in maize (or soybean) with N application, *N*_IM0_ (or *N*_IS0_) is the total N accumulation in maize (or soybean) without N application, and *N*_M_ (or *N*_S_) is the amount of N supplied during the growing season.

#### 2.3.7 Nitrogen agronomic efficiency

Nitrogen agronomic efficiency (AE_N_) reflects the increase in grain yield per unit of N applied. The AE_N_ was calculated with reference to Zhang et al. (2020b).


$${\text{AE}}_{\text{N}}=\frac{Y_{IM}\left(Y_{IS}\right)-Y_{IM0}\left(Y_{IS0}\right)}{N_{M}\left(N_{S}\right)}$$

Where *Y*_IM0_ (or *Y*_IS0_) refers to the yield of maize (or soybean) without fertilization in intercropping.

#### 2.3.8 Nitrogen partial factor productivity

Partial factor productivity (PFP) of N fertilizer is an index to measure the relationship between N input per unit area and crop yield per unit area. The PFP was calculated with reference to Liang et al. (2020).


$$\text{PFP}=\frac{Y_{IM}\left(Y_{IS}\right)}{N_{M}\left(N_{S}\right)}$$

#### 2.3.9 Economic benefit

Cost–profit analysis was performed following Li et al. (2022b):


Net return (NR)=Gross product−Total cost

$$\text{Gross product}=Product_{M}+Product_{S}=Yield_{M}\times Price_{M}+Yield_{S}\times Price_{S}\;$$

Total cost=cost(fertilizer input)+cost(seed)+cost(other)

Where Price_M_ and Price_S_ refer to the real-time purchase prices of maize and soybean by grain merchants, respectively. Cost (other) refers to the total cost of the lease (land), pesticide, and field management (plowing, harrowing, sowing, and harvesting) throughout the whole growth period of maize and soybean.

### 2.4 Statistical analysis

All the data in this study were statistically analyzed using SPSS statistical software (SPSS Inc., Chicago, IL, USA). One-way ANOVA was used to compare the response of maize and soybean yield, yield components, NR, leaf area index (LAI), AYL, LER, dry matter and N accumulation, and NUE to planting density and N input. The least significant difference (LSD) test was used to assess the difference between treatments at the *p* < 0.05 level. In addition, the correlation between yield components and yield was analyzed using linear regression analysis, with direct and indirect path coefficients calculated. Graphics were created using Origin 2021, Adobe Illustrator 2023, and BioRender.com.

## 3 Results

### 3.1 Yield and path analysis

The grain number, grain weight, and yield of maize and soybean were influenced by planting density (**Table 1**). Significant differences in yield were observed between years and treatments. The average yield of soybean did not show a significant difference over the 2-year test period, while the average yield of maize in 2023 was higher than in 2022. For the same soybean planting density, increasing maize density showed an increasing trend in maize yield. Specifically, the average yield of maize in D3 treatment increased by 18.5% and 34.9% compared to the D4 and D5 treatments, respectively. Under the same maize density, increasing soybean density also increased soybean yield. The soybean yield in D3 treatment increased by 30.1% and 18.2% compared to the D1 and D2 treatments, respectively. Considering different density combinations, the total yield of the intercropping system showed D3 > D2 > D1 > D4 > D5, with these trends consistent in both 2022 and 2023.

**Table 1:** Effects of different density treatments on the yield and yield components of soybean and maize.

Year (Y)	Density (D)	Maize yield components			Yield_M_ (kg ha^−1^)	Soybean yield components			Yield_S_ (kg ha^−1^)	Total yield (kg ha^−1^)
		EP (× 10^4^ plant)	GN (*n*)	TKW (g)		EP (× 10^4^ plant)	GN (*n*)	HKW (g)		
2022	D1	6.534	524.4 b	315.5 ab	10,808.9 a	6.446	117.3 c	24.4 a	1,907.4 c	12,716.3 ab
	D2	6.534	550.0 a	310.6 b	11,162.1 a	7.001	131.3 b	21.4 c	2,045.4 bc	13,207.6 a
	D3	6.534	518.4 b	313.4 b	10,617.3 a	8.556	141.7 a	24.1 a	2,796.0 a	13,413.3 a
	D4	5.784	499.3 c	320.8 a	9,262.5 b	8.556	118.7 c	21.5 c	2,259.1 b	11,521.5 b
	D5	5.034	474.7 c	322.0 a	7,691.2 c	8.556	134.3 ab	22.8 b	2,713.5 a	10,404.7 c
2023	D1	6.411	549.0 b	331.0 a	11,651.9 a	9.413	93.7 a	22.4 b	2,047.4 b	13,699.3 b
	D2	6.411	560.4 ab	337.1 a	11,958.5 a	10.598	95.0 a	22.4 b	2,333.8 a	14,292.2 a
	D3	6.411	578.8 a	326.1 ab	12,099.5 a	11.339	85.0 b	23.3 a	2,328.1 a	14,427.6 a
	D4	5.706	543.8 b	318.7 b	9,888.7 b	11.339	74.0 c	21.4 c	1,853.1 c	11,741.8 c
	D5	5.040	561.3 ab	324.7 ab	9,186.4 b	11.339	89.0 ab	23.1 a	2,402.3 a	11,588.8 c
ANOVA (*F*-value)										
Y		–	6.9^*^	141.0^**^	18.2^**^	–	46.1^**^	0.4	1.8	6.0^*^
D		–	0.7^*^	3.4^*^	24.6^**^	–	1.1^*^	8.9^**^	4.8^*^	10.3^**^
Y × D		–	0.6	26.2^**^	0.5	–	0.6	3.6 ^*^	1.8	0.2

The yield data of monoculture maize and monoculture soybean are detailed in **Supplementary Table S1** and were not analyzed separately. D1, D2, D3, D4, and D5 represent maize (soybean) planting densities of 6.75 × 10^4^ (9.75 × 10^4^) plants ha^−1^, 6.75 × 10^4^ (12 × 10^4^) plants ha^−1^, 6.75 × 10^4^ (14.25 × 10^4^) plants ha^−1^, 6.0 × 10^4^ (14.25 × 10^4^) plants ha^−1^, and 5.25 × 10^4^ (14.25 × 10^4^) plants ha^−1^, respectively. Values are means of three replicates. Different lowercase letters indicate different differences in treatment levels (LSD, p < 0.05). ^*^p < 0.05 and ^**^p < 0.01—levels of significant differences.

**Figure 3:**
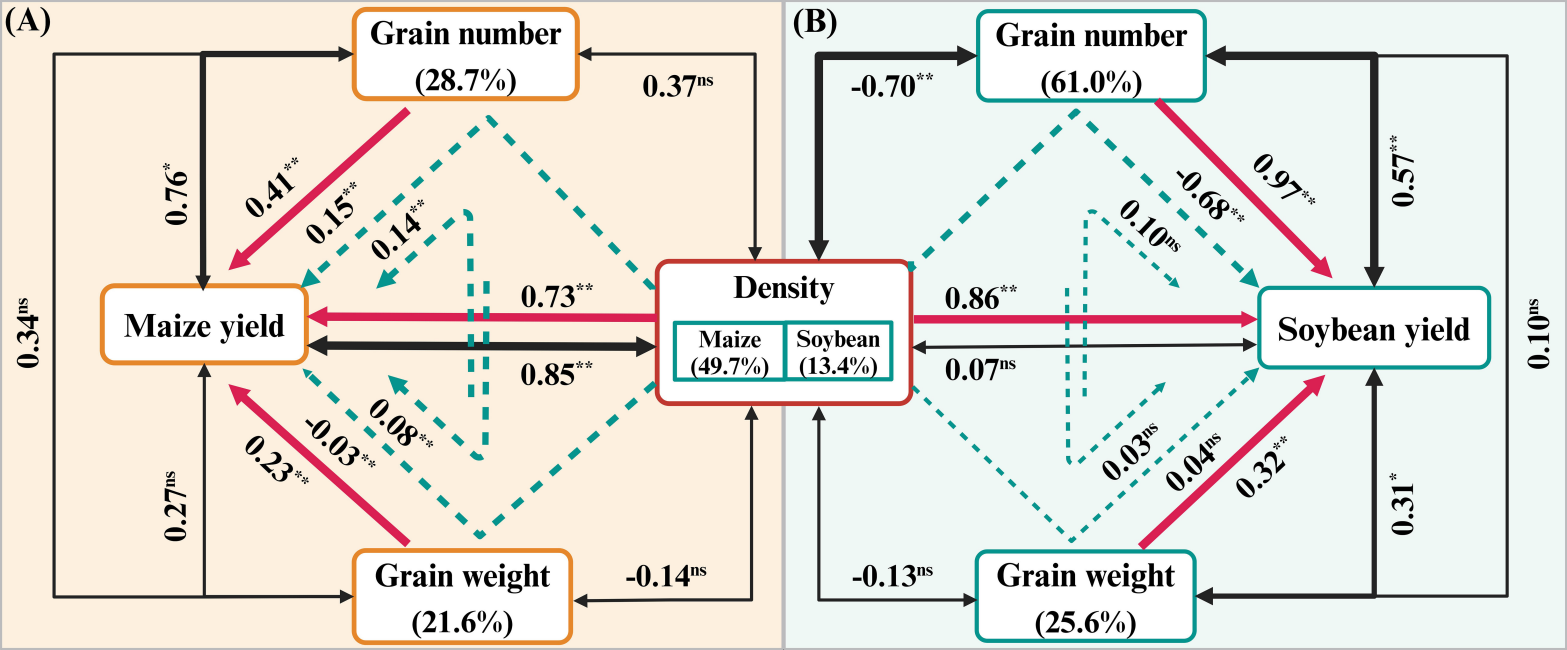
Path analysis of factors influencing yield components in the maize **(A)** and soybean **(B)** intercropping system. Path analysis was performed using the formula: 2 years × 5 treatments × 3 replicates = 30 sets of data pairs. The red solid single-arrow lines represent the direct effects of yield components on yield, while the blue dotted single-arrow lines represent the indirect effects. The black solid two-way arrow lines represent the correlations between yield components and yield. The data above the lines represent the direct path coefficients, indirect path coefficients, and correlation coefficients, respectively. The contribution rate of density, grain number, and grain weight to yield was calculated as the ratio of the sum of the effects of each individual factor (including direct and indirect effects) to the sum of the effects of all factors, expressed as a percentage in parentheses. ns, insignificant differences, while the asterisks (^*^ and ^**^) represent the significant differences at *p* < 0.05 and *p* < 0.01 levels, respectively, for each coefficient. The thickness of the lines reflects the size of the significance.

The path analysis reflected the effects of yield components on the yield of maize and soybean (**Figure 3**). Under different densities, the direct effect of yield components on yield was greater than the indirect effect. The number of ears and grains per ear were significantly positively correlated with yield. For maize yield, the direct positive effects of yield components were as follows: density (0.73^**^) > grain number (0.41^**^) > grain weight (0.23^**^), with corresponding contribution rates of 49.7%, 28.7%, and 21.6%, respectively. The direct positive effects of yield components on soybean yield were as follows: grain number (0.97^**^) > density (0.86^**^) > grain weight (0.32^**^), with corresponding contribution rates of 61.0%, 13.4%, and 25.6%, respectively.

### 3.2 Economic benefit analysis

Significant differences were observed in the gross product value and net return of the intercropping system under a combination of different densities (**Table 2**). Affected by the market situation of maize and soybean, year and treatment had significant effects on net return, and the difference in total cost was mainly due to variations in fertilizer and seed inputs. Over the 2 years of the density test, the total output value and net income of D3 treatment were the largest, being 16.4% (D1), 6.7% (D2), 45.2% (D4), and 49.1% (D5) higher than other treatments, respectively.

**Table 2:** Economic benefit analysis (CNY ha^−1^) for 2022 and 2023.

Year (Y)	Density (D)	Cost (fertilizer input)		Cost (seed)	Cost (other)				Total input		Product_M_	Product_S_		Gross product	Net return (NR)
					Lease	Pesticide		Field management							
2022	D1	2,775		1,215	7,500	420		8,100	20,010		30,265 ab	11,063 c		41,328 a	21,318 ab
	D2	2,775		1,305	7,500	420		8,100	20,100		31,254 a	11,864 bc		43,118 a	23,018 a
	D3	2,775		1,350	7,500	420		8,100	20,145		29,728 ab	16,217 a		45,945 a	25,800 a
	D4	2,775		1,275	7,500	420		8,100	20,070		25,935 bc	13,103 b		39,038 b	18,968 b
	D5	2,775		1,410	7,500	420		8,100	20,205		21,535 c	15,738 a		37,274 b	17,069 b
2023	D1	2,775		1,215	7,500	420		8,100	20,010		30,295 a	10,032 ab		40,327 ab	20,317 a
	D2	2,775		1,305	7,500	420		8,100	20,100		31,092 a	11,435 ab		42,527 a	22,427 a
	D3	2,775		1,350	7,500	420		8,100	20,145		31,459 a	11,408 ab		42,867 a	22,722 a
	D4	2,775		1,275	7,500	420		8,100	20,070		25,711 b	9,080 b		34,791 b	14,721 b
	D5	2,775		1,410	7,500	420		8,100	20,205		23,885 b	11,711 a		35,656 b	15,451 b
ANOVA (*F*-value)															
				Product_M_	Product_S_				Gross product					NR	
Y				0.7	23.4^**^				3.9^*^					3.9^*^	
D				14.0^**^	4.8^*^				6.9^**^					7.0^**^	
Y × D				0.4	2.0				0.3					0.3	

D1, D2, D3, D4, and D5 represent maize (soybean) planting densities of 6.75 × 10^4^ (9.75 × 10^4^) plants ha^−1^, 6.75 × 10^4^ (12 × 10^4^) plants ha^−1^, 6.75 × 10^4^ (14.25 × 10^4^) plants ha^−1^, 6.0 × 10^4^ (14.25 × 10^4^) plants ha^−1^, and 5.25 × 10^4^ (14.25 × 10^4^) plants ha^−1^, respectively. Price_M_ was 2.8 CNY kg^−1^ and 2.6 CNY kg^−1^ in 2022 and 2023, respectively, and Price_S_ was 5.8 CNY kg^−1^ and 4.9 CNY kg^−1^ in 2022 and 2023, respectively. The price of slow-released compound fertilizer was 2.3 CNY kg^−1^, and the price of compound fertilizer was 2 CNY kg^−1^. Pesticide costs, including herbicides and insecticides, were 195 CNY ha^−1^ and 225 CNY ha^−1^, respectively. The cost of field management, which includes plowing, harrowing, sowing, and harvesting, were 1,500 CNY ha^−1^, 1,500 CNY ha^−1^, 2,100 CNY ha^−1^, and 3,000 CNY ha^−1^, respectively. Gross product and NR values are means of three replicates. Different lowercase letters indicate differences (LSD, *p* < 0.05). ^*^ *p* < 0.05 and ^**^ *p* < 0.01—levels of significant differences in NR.

### 3.3 Land equivalent ratio and actual yield loss index

**Figure 4:**
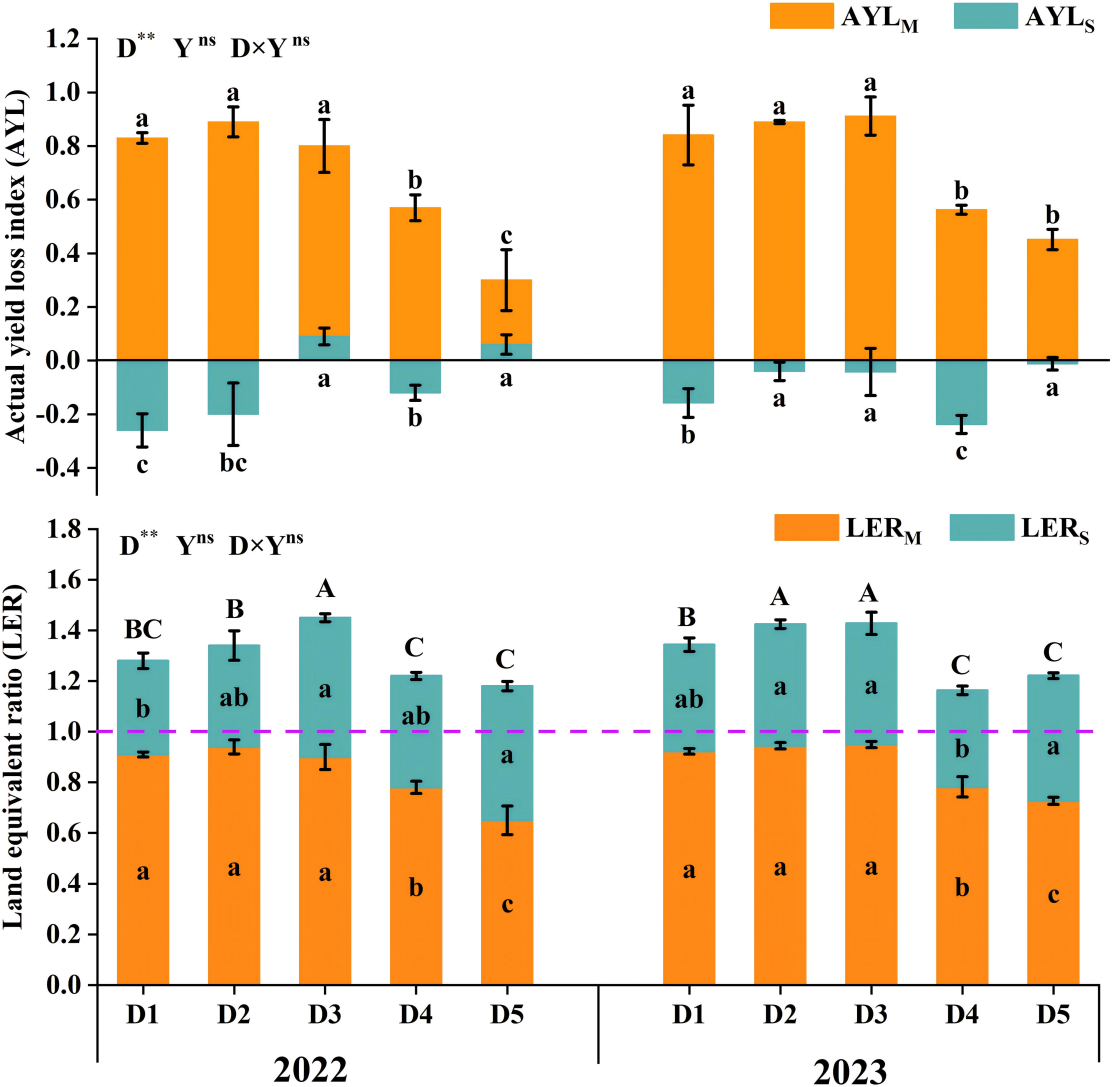
Effects of density allocation and N input on the land equivalent ratio (LER) and actual yield loss index in the maize and soybean intercropping system. D1, D2, D3, D4, and D5 represent maize (soybean) planting densities of 6.75 × 10^4^ (9.75 × 10^4^) plants ha^−1^, 6.75 × 10^4^ (12 × 10^4^) plants ha^−1^, 6.75 × 10^4^ (14.25 × 10^4^) plants ha^−1^, 6.0 × 10^4^ (14.25 × 10^4^) plants ha^−1^, and 5.25 × 10^4^ (14.25 × 10^4^) plants ha^−1^, respectively. The values are means of three replicates. Lowercase letters in the columns represent significant levels of AYL_X_ and PLER under different treatments (LSD, *p* < 0.05), while uppercase letters represent significant differences in LER at *p* < 0.05 level. The purple dotted line represents the case where the LER value is 1. ns, insignificant difference; the asterisks ^**^ represent the significant differences at *p <*0.01 levels, respectively.

The AYL indexes of maize under different density treatments were all greater than 0 (AYL_M_ > 0). Among them, the AYL indexes of soybean under the D3 and D5 treatments in 2022 were also higher than 0 (AYL_S_ > 0), while the AYL indexes for soybean under other treatments were all less than 0 (AYL_S_ < 0). This indicates that intercropping maize provides a significant yield advantage across different density fertilizer configurations, and that the relative yield of intercropped soybean under suitable density fertilizer configurations could be greater than that of monoculture soybean (**Figure 4**). The results of the 2-year experiment also showed that the dense fertilizer configuration significantly affected AYL. The AYL of density treatments followed the trend D3 > D2 > D1 > D4 > D5, consistently over the 2 years.

The LER under different N treatments was greater than 1 (**Figure 4**), and the LER_M_ was greater than LER_S_. This indicates that maize–soybean intercropping had significant yield advantages and significantly improved land use efficiency. The yield advantage of maize in intercropping was greater than that of soybean. In addition, the results also showed that the density configuration significantly affected LER, with no significant difference between years. The largest LER was observed in the D3 density.

### 3.4 Leaf area index

**Figure 5:**
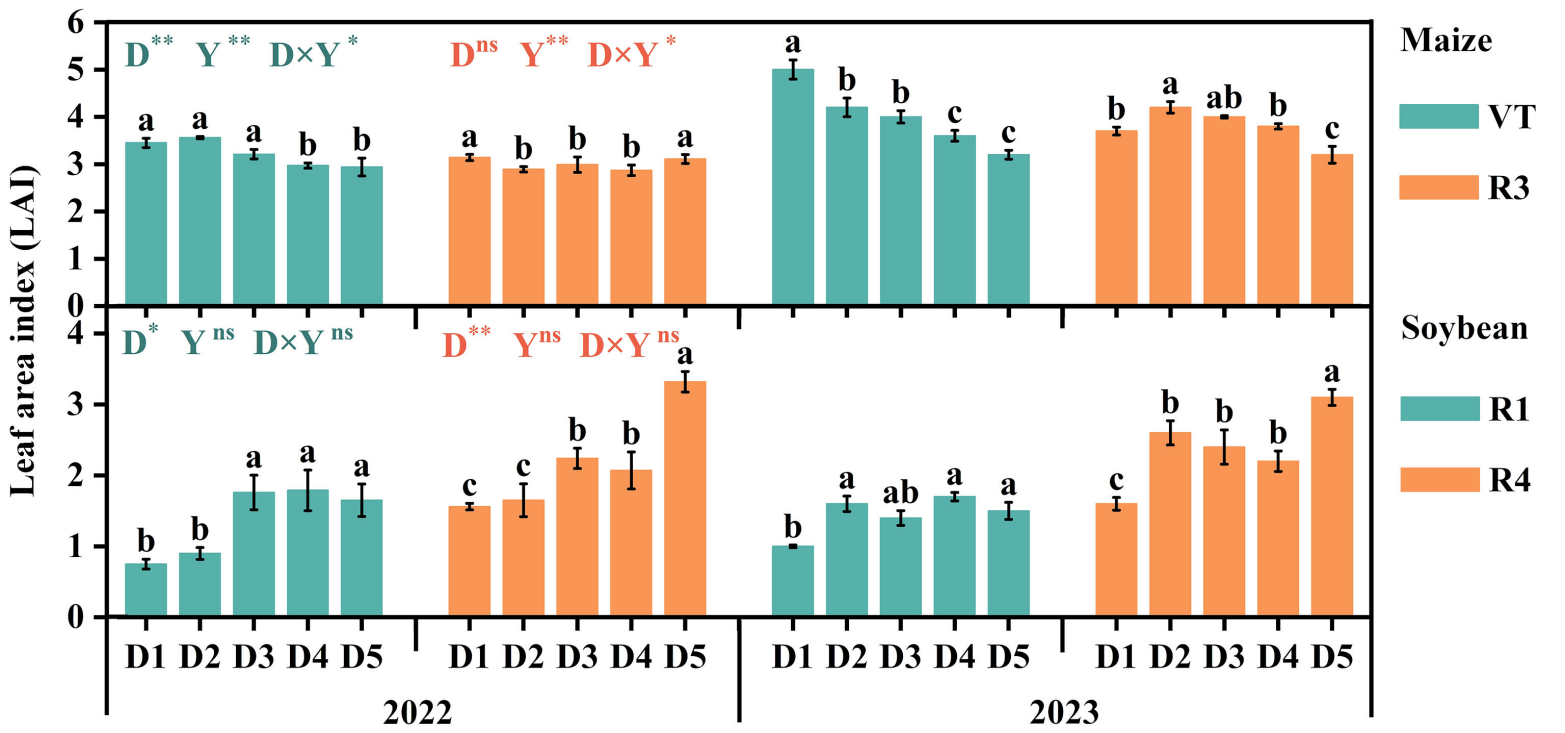
Effects of density configuration on leaf area index (LAI) in the maize and soybean intercropping system. Top panel (maize): VT, the silking stage; R3, the milk stage. Bottom panel (soybean): R1, the first flowering date stage; R4, the beginning pod stage. D1, D2, D3, D4, and D5 represent maize (soybean) planting densities of 6.75 × 10^4^ (9.75 × 10^4^) plants ha^−1^, 6.75 × 10^4^ (12 × 10^4^) plants ha^−1^, 6.75 × 10^4^ (14.25 × 10^4^) plants ha^−1^, 6.0 × 10^4^ (14.25 × 10^4^) plants ha^−1^, and 5.25 × 10^4^ (14.25 × 10^4^) plants ha^−1^, respectively. Values are means of three replicates. Lowercase letters above the columns indicate the differences between different treatments (LSD, *p* < 0.05). Results of variance analysis for multiple comparisons in maize (soybean) at VT (R1) and R3 (R4) are distinguished by cyan and orange, respectively. ns, insignificant differences; the asterisks (^*^ and ^**^) represent the significant differences at *p* < 0.05 and *p* < 0.01 levels, respectively.

Significant differences in LAI were observed between maize and soybean under different densities (**Figure 5**). The LAI of maize in 2023 was greater than in 2022, while there was no significant difference in the LAI of soybean between the 2 years. Maize exhibited significant differences at VT, with D2 (2022) and D1 (2023) being the largest. The LAI of soybean at R4 was higher than R1, with D3 having the highest LAI at R1. Additionally, the LAI of D5 was significantly higher than other treatments at R4. Overall, the LAI in 2023 was greater than in 2022.

### 3.5 Dry matter accumulation and distribution

**Figure 6:**
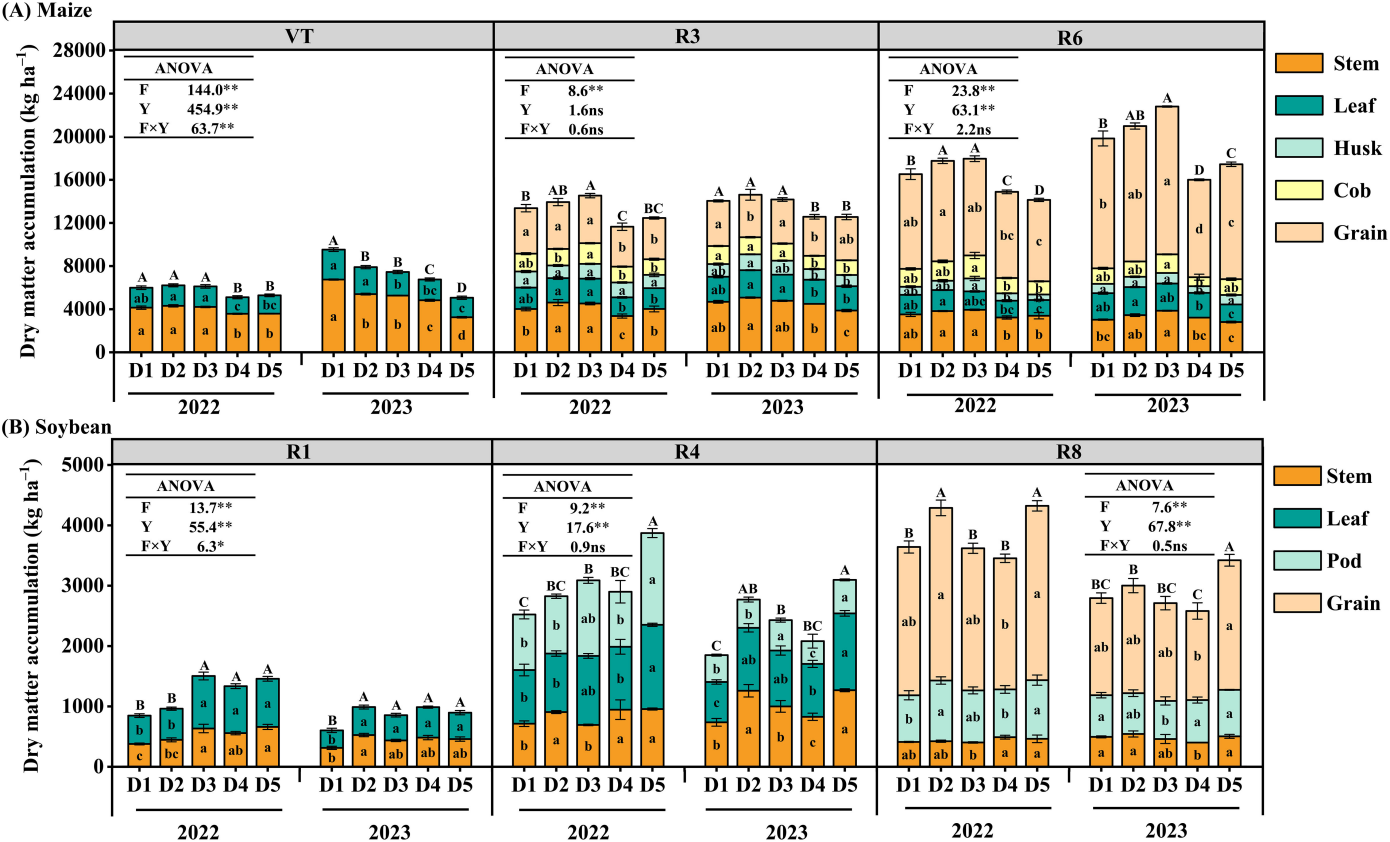
Effects of planting density on dry matter accumulation in the maize **(A)** and soybean **(B)** intercropping system. D1, D2, D3, D4, and D5 represent maize (soybean) planting densities of 6.75 × 10^4^ (9.75 × 10^4^) plants ha^−1^, 6.75 × 10^4^ (12 × 10^4^) plants ha^−1^, 6.75 × 10^4^ (14.25 × 10^4^) plants ha^−1^, 6.0 × 10^4^ (14.25 × 10^4^) plants ha^−1^, and 5.25 × 10^4^ (14.25 × 10^4^) plants ha^−1^, respectively. Values are means of three replicates. Different lowercase letters in the columns indicate the significant differences in dry matter accumulation in each organ of maize (or soybean) under different treatments (LSD, *p* < 0.05), while different uppercase letters indicate the significant differences in dry matter accumulation in the whole plant of maize (or soybean) under different treatments at the *p* < 0.05 level. ns, insignificant differences; the asterisks (^*^ and ^**^) represent significant differences at the *p* < 0.05 and *p* < 0.01 levels, respectively.

Different densities affected the dry matter accumulation of maize and soybean populations. In 2023, dry matter accumulation of maize was higher than in 2022, whereas soybean dry matter accumulation decreased (**Figure 6**). The dry matter accumulation of maize increased with increasing density, with D3 showing significantly higher accumulation than other treatments at VT, R3, and R6 (**Figure 6A**). At R6, the average accumulation of D3 increased by 11.7% (D1), 4.9% (D2), 31.6% (D4), and 28.8% (D5), respectively. The dry matter accumulation of soybean also increased with increasing density, except during the R1 period in 2022 and 2023. Among the treatments, D5 had the largest dry matter accumulation (**Figure 6B**). The average accumulation of D3 increased by 20.5% (D1), 7.4% (D2), 22.8% (D3), 28.9% (D4), respectively, at R8. In addition, different density configurations have a significant effect on the dry matter accumulation of each organ of maize and soybean (**Figure 6**). The increase in maize density promoted a higher grain dry matter distribution ratio at the R6 stage in both years. Specifically, grain dry matter enrichment was 32.0% and 23.7% higher in the D3 treatment than in the D4 and D5 treatments, respectively. For soybean, the dry matter accumulation of various organs did not increase with increasing density; however, the D2 treatment exhibited the highest proportion of grain dry matter distribution at the R8 stage. Variance analysis showed that the dry matter accumulation of maize and soybean was affected by both density fertilizer treatment and year. In addition, the interaction between density and year was significant at the maize VT stage and the soybean R1 stage.

### 3.6 Nitrogen accumulation and distribution

**Figure 7:**
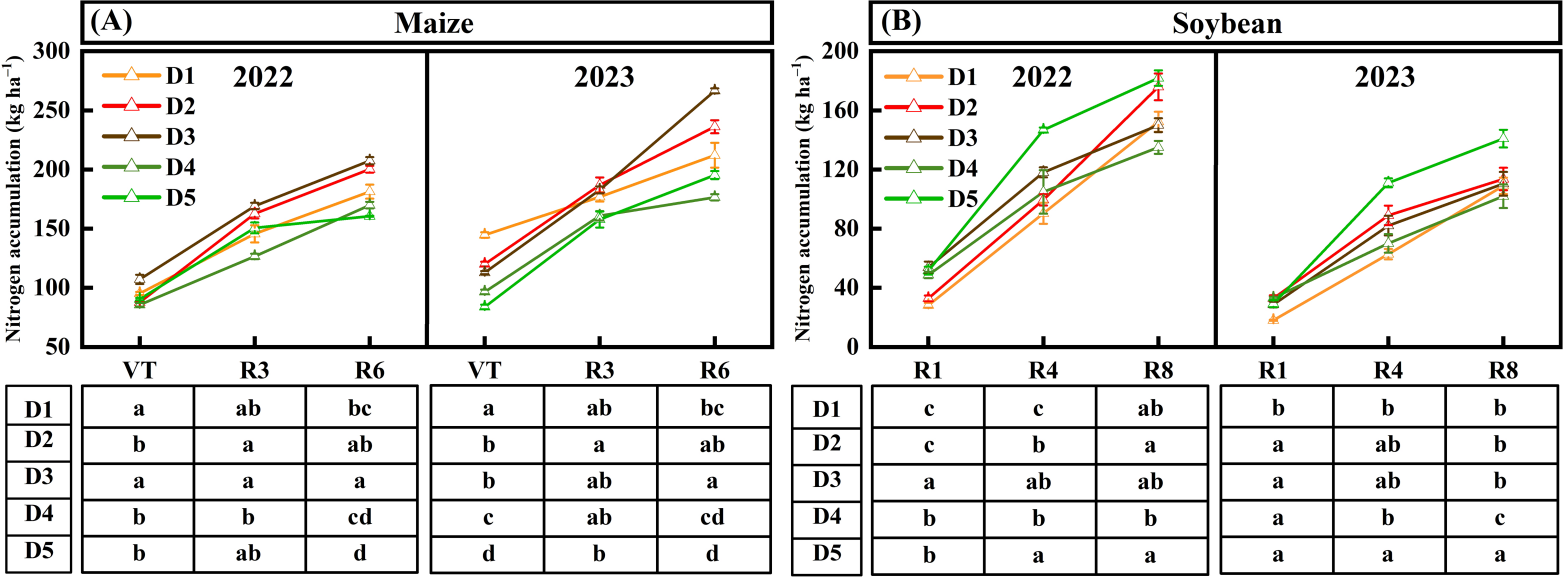
Effects of planting density on N accumulation in the maize **(A)** and soybean **(B)** intercropping system. D1, D2, D3, D4, and D5 represent maize (soybean) planting densities of 6.75 × 10^4^ (9.75 × 10^4^) plants ha^−1^, 6.75 × 10^4^ (12 × 10^4^) plants ha^−1^, 6.75 × 10^4^ (14.25 × 10^4^) plants ha^−1^, 6.0 × 10^4^ (14.25 × 10^4^) plants ha^−1^, and 5.25 × 10^4^ (14.25 × 10^4^) plants ha^−1^, respectively. Values are means of three replicates. Different lowercase letters in the table below the line chart indicate significant differences in N accumulation at each stage of maize (or soybean) under different treatments (LSD, *p* < 0.05).

The N accumulation of maize and soybean varied significantly different under different density treatments at each growth stage (**Figure 7**). Increasing density of maize and soybean increased the population N accumulation. N accumulation of maize in D3 was higher than in other treatments. The N accumulation of D3 treatment increased by 20.0% (D1), 8.2% (D2), 36.4% (D4), and 32.8% (D5), respectively, at R6. The N accumulation of soybean in D5 was the highest, which increased by 24.3% (D1), 13.7% (D2), 24.4% (D3), and 36.4% (D4), respectively, at R8.

### 3.7 Nitrogen use efficiency

**Table 3** demonstrates the significant effects of different densities on the N uptake efficiency of maize and soybean. The 2-year experiment results revealed that increasing maize planting density notably enhanced the AE_N_, PFP, and NUE of maize. The increasing density of soybeans also increased the NUE. Additionally, the AE_N_ and PFP of soybeans also increased in 2022. However, no significant differences were observed in 2023. Among all density treatments, the NUE, AE_N_, and PFP were significantly higher in the D3 treatment compared to others. Variance analysis showed that both the year and treatment significantly affected the NUE and PFP of maize. The AE_N_ and PFP of soybean were only affected by treatments, with no significant differences observed between years. At the same time, the AE_N_ of maize only showed differences between treatments.

**Table 3:** Effects of different density treatments on N efficiency in maize and soybean.

Year (Y)	Density (D)	NUE (%)	AE_N_ (kg kg^−1^)		PFP (kg kg^−1^)	
			Maize	Soybean	Maize	Soybean
2022	D1	35.2 b	15.2 a	11.9 b	42.4 a	42.4 b
	D2	49.4 a	16.5 a	14.9 b	43.8 a	45.5 b
	D3	43.1 ab	14.4 a	31.6 a	41.6 a	62.1 a
	D4	25.7 c	9.1 b	19.7 b	36.3 ab	50.2 ab
	D5	38.2 b	2.9 c	29.8 a	30.2 b	60.3 a
2023	D1	21.7 b	12.4 a	12.1 ab	45.7 a	45.5 ab
	D2	31.3 b	13.6 a	18.4 a	46.9 a	51.9 a
	D3	40.3 a	14.1 a	18.3 a	47.4 a	51.7 a
	D4	7.5 c	5.5 b	7.8 b	38.8 ab	41.2 b
	D5	26.7 b	2.7 b	20.0 a	36.0 b	53.4 a
ANOVA (*F*-value)						
Y		35.9^**^	1.4	3.3	9.4^*^	1.9
D		18.1^**^	12.0^**^	5.7^*^	12.0^**^	4.1^*^
Y × D		1.7	0.4	2.1	0.3	1.8

D1, D2, D3, D4, and D5 represent maize (soybean) planting densities of 6.75 × 10^4^ (9.75 × 10^4^) plants ha^−1^, 6.75 × 10^4^ (12 × 10^4^) plants ha^−1^, 6.75 × 10^4^ (14.25 × 10^4^) plants ha^−1^, 6.0 × 10^4^ (14.25 × 10^4^) plants ha^−1^, and 5.25 × 10^4^ (14.25 × 10^4^) plants ha^−1^, respectively. Different lowercase letters indicate differences in different treatments (LSD, *p* < 0.05). ^*^ *p* < 0.05 and ^**^ *P*<0.01—levels significant differences.

## 4 Discussion

### 4.1 Yield and economic benefits

Optimizing planting density is a crucial cultivation measure for enhancing crop yield (Ren et al., 2016; Yang et al., 2024). Previous research on MSSI has primarily concentrated on variations in main crop (maize) density (Raza et al., 2022; Yang et al., 2022). Adjusting maize density in MSSI has been shown to enhance grain yield and economic returns (Ren et al., 2016). Our study further validated these findings and observed notable variations in yield and net income across different combinations of density. The combination of the lowest maize density resulted in the lowest grain yield and net return. However, increasing maize density appropriately led to higher maize yield and net return (**Table 1**), while no effect from soybean density. This maybe because maize is the dominant high-yielding crop in MSSI, whereas soybean is a lower-yielding crop. Maize can achieve yields close to those of single-cultivation systems, while soybean yields only reach about 60% of those obtained with single cultivation (Zhang et al., 2021; Yang et al., 2024).

Previous research has indicated that the increase in maize yield in intercropping is attributed to the increase of effective ears per unit area, while the decrease in soybean yield is primarily linked to a reduction in grain number per plant (Koesmaryono and Sugimoto, 2005; Huang et al., 2019; Fan et al., 2020). Our path analysis results further confirmed this perspective. Density exhibited the strongest and most significant positive correlation and direct positive impact on maize yield, whereas grain number exhibited the strongest and most significant positive correlation with soybean yield and a direct positive effect (**Figure 3**). Other studies have shown that soybean density and grain number exhibit was alternating growth and decline, resulting in a small difference in yield between low and high densities (Wang et al., 2023a). In this study, soybean density was significantly negatively correlated with grain number, and their combined indirect effect on yield was also negative, consistent with previous research (Wang et al., 2023a). In addition, the results of yield contribution rate further reflected the influence of density and grain number on maize and soybean, respectively. This means that it may be more meaningful to focus on the planting density of maize and the mechanisms to increase the number of soybean grains in order to achieve higher planting efficiency in MSSI.

### 4.2 Land equivalent ratio and actual yield loss index

LER is a commonly used indicator for measuring the yield advantage of intercropping. A recent meta-analysis revealed that the overall LER for MSSI in China was 1.6 (Yang et al., 2024). Our results were lower, with average LER values reaching only 1.30 and 1.32 in 2022 and 2023, respectively. However, these values still demonstrate a significant yield advantage for MSSI. Previous research indicated that increasing maize density under the same soybean density in MSSI tends to reduce LER (Echarte et al., 2011; Liu et al., 2023). However, our study found that increasing the density of the other crop, while keeping soybean or maize density constant, improved LER (**Figure 4**). The AYL more accurately reflects competition between and within crops in MSSI, with positive or negative AYL_X_ values indicating yield gains or losses (Liu et al., 2023; Mohammadkhani et al., 2023). In our study, AYL_M_ values under all treatments showed similar results (AYL_M_ > 0). We also found that the competitive advantage of soybeans under high density was improved, which was reflected in the D3 treatment in 2022 (**Figure 4**).

### 4.3 Leaf area index and dry matter accumulation

In this study, increasing maize density significantly increased LAI and dry matter accumulation, which was consistent with previous results (Ren et al., 2016; Xia et al., 2019). Normally, sufficient leaf area provides more light resources for plants, improving their biosynthetic ability, and a higher LAI typically results in higher total dry matter accumulation (Wu et al., 2018). However, our study found limited support for this conclusion, particularly regarding soybean plants. The LAI of soybean at R1 and R4 exhibited higher values in certain treatments (D2) in 2023 compared to 2022. Surprisingly, the dry matter accumulation at growth stage R8 was lower in 2023 under the same treatment. Furthermore, the total dry matter accumulation of maize grains and whole plants was higher in 2023 than in 2022, whereas soybeans displayed the opposite trend. This may be attributed to significantly higher precipitation during the experimental period in 2023 compared to 2022, which affected the normal growth and development of maize and soybeans and limited the synthesis of plant biomass (Raza et al., 2020; Li et al., 2023c).

### 4.4 Nitrogen accumulation and utilization efficiency

This study observed the dynamic changes in N accumulation in maize and soybean at three crucial growth stages (**Figure 7**). Previous research has shown that optimizing crop density in intercropping enhances N accumulation (Fan et al., 2019). We further found that adjusting maize density also influences N accumulation in soybean. N accumulation of soybean under the D5 treatment during the 2-year experiment exhibited a consistent trend with dry matter accumulation, surpassing other treatments significantly (**Figure 6**). High-position crops (maize) in MSSI exerted shading stress on low-position crops (soybean), and reducing the density of maize enhanced the light environment for soybean (Yang et al., 2015). Therefore, the imoroved light environment promoted the soybean with D5 treatment to achieve the largest LAI and grain yield, resulting in the highest N accumulation. However, shading stress from maize on soybean typically occurs after soybean enters the R1 stage, as maize transitions from vegetative to reproductive stages later than soybean (at this time, maize has not yet formed the maximum LAI and population biomass) (Fan et al., 2018; Li et al., 2023b). Our study supports the finding that the N accumulation at the R1 stage of soybean was not significantly different across all treatments.

The effectiveness of MSSI in enhancing the absorption and utilization efficiency of N fertilizer has been validated in various studies (Chen et al., 2017; Nasar et al., 2023). In this research, three indicators (NUE, AE_N_, and PFP) were used to observe the N use efficiency of the intercropping system and the nitrogen agronomic efficiency and productivity of maize and soybean with nitrogen fertilizer (**Table 3**). With appropriate increases in plant density, NUE tended to improve (D3 > D4 in both years, D3 > D2 > D1 in 2023). Additionally, maize exhibited higher AE_N_ under D3 treatment, while soybean showed higher AE_N_ and PFP under the same treatment. Nowadays, it is generally believed that, based on the original soil fertility, improving N absorption and productivity of crops per unit area is a necessary measure for achieving higher yields (Spiertz, 2010). As shown in **Table 1**, the D3 treatment resulted in the highest total grain yield in both experiments.

## 5 Conclusions

A 2-year maize–soybean strip intercropping experiment showed that increasing density could significantly increase intercropping productivity. Due to the increase in leaf area index, the accumulation of dry matter and N was promoted, improving the utilization efficiency of N fertilizer. Consequently, the grain yield, land equivalent ratio, and economic benefit were improved. Path analysis showed that grain number and density were the most significant factors for grain yield in MSSI. In summary, optimizing the density of the two crops in MSSI can comprehensively optimize the yield formation mechanism within the intercropping population, thereby maximizing the yield-increasing potential of MSSI. In this study, the average maize (soybean) harvest density of 64,725 plants ha^−1^ (99,475 plants ha^−1^) was identified as the optimal combination of density. This provided an updated field configuration for the future continuous adoption of the MSSI mode in the summer maize planting areas of Huang-Huai-Hai and the Southern area of China.

## Data Availability

The original contributions presented in the study are included in the article/**Supplementary Material**. Further inquiries can be directed to the corresponding author.
