# Supplementary material for: Enhancing production efficiency through optimizing plant density in maize–soybean strip intercropping
Source: Front Plant Sci. 2024 Oct 11;15:1473786. doi: 10.3389/fpls.2024.1473786 (PMC11505130; doi:10.3389/fpls.2024.1473786)
Supplement: Supplementary file 1 [file Table1.docx]

Table.1S. Yield of monoculture maize and soybean under different fertilization inputs

| Year | N input (kg ha^-1^) | Maize | | | | N input (kg ha^-1^) | Soybean | | | |
| --- | --- | --- | --- | --- | --- | --- | --- | --- | --- | --- |
|  |  | EP  (×10^4^ plant ha^-1^) | GN  (n) | TKW  (g) | Yield  kg ha^-1^ |  | EP  (×10^4^ plant ha^-1^) | GN  (n) | HKW  (g) | Yield  kg ha^-1^ |
| 2022 | 0 | 6.722 | 441 b | 281.3 b | 8338.7 b | 0 | 14.250 | 63 b | 20.6 b | 1906.2 b |
|  | 255 | 6.722 | 545 a | 322.9 a | 11828.4 a | 45 | 14.250 | 157 a | 22.2 a | 5126.2 a |
| 2023 | 0 | 6.560 | 456 b | 280.4 b | 8369.3 b | 0 | 15.132 | 61 b | 21.6 b | 2062.2 b |
|  | 255 | 6.560 | 574 a | 336.1 a | 12659.1 a | 45 | 15.132 | 130 ab | 23.9 a | 4862.6 a |

EP, the effective plant population per hectare for maize or soybeans. GN, the number of grains per plant of maize or soybean. TKW, the weight of 1000 maize kernels (with a moisture content of 14%). HKW, the weight of 100 soybean grains (with a moisture content of 13%).
